# Prevalence of Anemia and Associated Factors among Secondary School Adolescent Girls in Jimma Town, Oromia Regional State, Southwest Ethiopia

**DOI:** 10.1155/2020/5043646

**Published:** 2020-09-22

**Authors:** Kelemu Fentie, Tolassa Wakayo, Getu Gizaw

**Affiliations:** ^1^Jimma University Medical Center, Jimma, Ethiopia; ^2^Department of Human Nutrition and Dietetics, Faculty of Public Health, Institute of Health, Jimma University, Jimma, Ethiopia

## Abstract

**Background:**

Anemia defined as a low blood hemoglobin concentration is public health importance. The adolescence age group is the most neglected in public health and nutrition research as priorities are usually given to pregnant women, lactating mothers, and their children less than 2 years. Current Ethiopian Food and Nutrition policy included adolescent girls in the most at-risk group for nutritional demands; however, only a few published studies have assessed a deficiency of anemia and associated factors to tackle the intergenerational cycle of malnutrition.

**Objective:**

To assess the prevalence of anemia and associated factors among high school adolescent girls in Jimma town.

**Methods:**

Data were collected from 528 secondary school adolescent girls by a school-based cross-sectional study design in Jimma town from 1/1/2019 to 1/2/2019, southwest Ethiopia. A multistage sampling technique was used to select the study participants. A portable battery-operated HemoCue Hb 301+ analyzer was used to measure the hemoglobin level, and then reading was classified as normal Hb ≥ 12 g\dl and anemic if the hemoglobin value <12 g/dl based on the WHO 2011 recommended cutoff points after adjustments to altitude was made. Bivariate analysis at *p* value ≤0.25 was considered as a candidate for multivariable logistic regression. Multivariable logistic regression was done to control for confounders and to identify factors independently associated with anemia. Level of statistical significance was declared at *p* < 0.05.

**Results:**

A total of 528 adolescent girls were included in the study yielding a response rate of 95.8%. The prevalence of anemia was found to be 26.7%, 95% CI (22.7, 30.50). In multivariate logistic regression analysis, those living separately from their family (AOR = 4.430, 95% CI (2.20, 8.90)), low dietary diversity score (AOR = 3.57, 95% CI (1.88, 6.75)), menstrual bleeding more than 5 days (AOR = 2.25, 95% CI (1.17, 4.33)), and low economic status (AOR = 2.16, 95% CI (1.17, 4.33)) were positively associated factors with anemia and only having at least a secondary school in mother's educational status AOR = 0.43, 95% CI (0.18, 0.97) was negatively associated with anemia in the study area.

**Conclusion:**

Prevalence of anemia among school adolescent girls was moderate public health importance according to the World Health Organization prevalence estimation of anemia. The living condition of the adolescent girls, dietary diversity score, duration of menses, and low economic status were positive predictor variables, whereas mothers who are being secondary school and above was a protective factor for anemia. Therefore, iron-rich and diversified food consumption should be given attention.

## 1. Introduction

Adolescents are young people between the ages of 10 and 19 years [1]. More than 1.2 billion adolescents are found in the world. The vast majority of adolescents (90%) live in low- or middle-income countries (LMICs) [2]. Adolescents and children constitute about 48% of the Ethiopian population, and about 25% of this age group is girls [3]. It is a period of rapid growth when up to 45% of skeletal growth takes place and 15 to 25% of adult height is achieved during this period [4]. During the growth spurt of adolescence, up to 37% of total bone mass may be accumulated. Although, nutrition influences growth and development throughout infancy, childhood, and adolescence, pieces of evidence show that nutrient needs including that of iron are the greatest during the period of adolescence [4].

Anemia, defined as a low blood hemoglobin concentration, is a public health problem that affects LMICs and has significant adverse health consequences including morbidity and mortality as well as adverse impacts on social and economic development [5]. Iron deficiency is the most prevalent nutritional deficiency and the most common cause of anemia in the world. It is characterized by a defect in hemoglobin synthesis, resulting in red blood cells that are abnormally small (microcytic) and a decreased amount of hemoglobin (hypochromic). Asia and Africa are regions with a higher prevalence of anemia. Nutritional deficiencies are regarded as the most important cause of anemia in the world and a major potential contributor to adolescent anemia in sub-Saharan Africa [6].

WHO defines anemia as a condition in which hemoglobin (Hb) content of blood is lower than normal as a result of deficiency of one or more essential nutrients. Based on WHO 2011, if the hemoglobin level is ≥12 g/dl, it does not indicate anemia for males and females of age between 12 and 14 years and for nonpregnant women >15 years. Anemia is established if the level of hemoglobin is <12 g/dl for nonpregnant women >15 years and children 12–14 years old and 11–11.9 g/dl, 8–10.9 g/dl, and 8 mg/dl were consider as having mild, moderate, and severe anemia respectively [7]. Based on the public health importance, if the prevalence of anemia ≤4.9% is no public health problem, 5.0–19.9% mild public health problem, 20.0–39.9 moderate public health problem, and ≥40 severe health problem [8].

The WHO estimates the prevalence of anemia among adolescent girls in southwest Asian countries like Indonesia, Nepal, and Bhutan was 30%, 46%, and 58.6%, respectively [9]. Similarly, in sub-Saharan Africa, about half of adolescent girls are anemic [5]. Local studies in Babile, eastern Ethiopia, were 32%, and this study concluded the nutritional status of adolescent girls contributes to the nutritional status of the community [10]. Another study conducted in the Afar region, Ethiopia, shows that the prevalence of anemia among school-going adolescent girls was 22.9%, and it was a moderate public health problem [11].

Globally, the prevalence of anemia had shown dramatic increment among women of nonpregnant reproductive age groups from 464 million in 2000 to 578 million in 2016. A condition persists in LMICs which reported the overall prevalence of anemia was over 35%. So, there is still a long road ahead to achieve the SDG 2030 targets anemia in adolescent girls. In Ethiopia, eighteen percent (17.7%) nonpregnant women aged 15 to 49 are anemic, of which Somali regional state has the highest prevalence of anemia (34.8%) followed by the Gambella region where 26.7% and 19% of reproductive age nonpregnant women were anemic in Oromia regional state [12, 13].

The associated factors of anemia among adolescent girls differ from study to study, like low dietary diversity score, living status of adolescents with either of the two parents, duration of menstruation, history of parasitic infestation, low socioeconomic status, household family size, inadequacy of dietary iron intake, drinking tea immediately after a meal, high consumption of whole wheat bread, and low consumption of vitamin C rich foods and molasses, parent's level of education, parasite infections, low BMI, being stunted, and underweight [10, 11, 14, 15].

The risk of anemia increases during adolescent years with the onset of menstruation and pregnancy. Iron loss from menstruation must be countered by further high iron intake for young women; the other is ever-increasing evidence that control anemia in pregnant women may be more easily achieved if satisfactory iron status can be ensured during adolescence [6, 16]. Most of the previous studies on anemia in Ethiopia were conducted on pregnant and lactating women and children. A few studies assessed anemia and its predictors among adolescent girls in the country. Some of the reasons why there are few studies done in this age groups are they are assumed as being less vulnerable to nutritional deficiency than the other groups which are not true, given the fact that adolescence exerts significantly increased demands on both micro- and macronutrients due to the rapid changes occurring in physical as well as in body composition particularly among ladies experiencing their menarche. So, this study aimed to assess the prevalence and factors associated with anemia among school adolescent girls in Jimma town secondary schools.

## 2. Materials and Methods

### 2.1. Study Area and Period

A school-based cross-sectional study was conducted from 1/1/2019 to 1/2/2019 among adolescent girls aged 14 to 19 years. Jimma, the study area, is located 347 km away from Addis Ababa which is the capital city of Ethiopia. The town has 14 secondary schools both private and government. A total of 5694 adolescent girls were attending secondary school in the town. One teaching specialized hospital, one primary hospital, 5 health centers, and numerous private clinics are giving health care services of the population of the town.

### 2.2. Study Population and Sampling Procedure

Adolescent girls who attended their education in Jimma town secondary schools, who were healthy, and lived in the study area for greater than six month, and nonpregnant adolescent girls during the study period were included in the study. The sample size was determined using single population proportion formula based on the following assumptions: estimated 32% prevalence (*p*) of anemia among adolescent girls in Babile district eastern, Ethiopia, 95% confidence interval (CI), margin of error (*d*) 5%, and design effect of 1.5. Secondly, factors associated with anemia among adolescent girls were considered to calculate the sample size from the previous literature, and finally, the largest sample size was used for this study which is 551.

Multistage sampling was used to select the study participants. First, five schools (30%) were randomly selected from 14 secondary schools found in Jimma town. Then, the total number of students was obtained from each school director office. The sample size was distributed among the selected schools proportionally based on the students' size in each school. Finally, the allocated sample size was selected from each school by using simple random sampling technique (computer generated random number) (Annex 1).

### 2.3. Data Collection Procedure and Instrument

A structured questionnaire, an anthropometric measurement for thinness and stunting, a standard Dietary Diversity Score (DDS) questionnaire from FAO 2010, knowledge related to anemia, and laboratory investigation for hemoglobin were used to collect the data. Sociodemographic and socioeconomic factors (age, marital status, occupational status of the parents, educational status of the parents, living status of the adolescents, parental condition (existence of both father and mother), ethnicity, religion, and wealth index), knowledge regarding anemia, health-related questionnaires (status of menarche, age of menarche, duration of menses, current status menses, frequency of changing sanitary pad, history of worm infestation, history of malaria infection, and taking deworm medicine), anthropometric measurement (to determine stunting and thinness), dietary pattern (meal frequency per day, DDs (using 24 h recall methods), and time and frequency of consumption of tea/coffee in the schools were addressed for adolescents.

Data were collected face-to-face by trained four diploma nurses using a pretested and structured “Afaan Oromo and Amharic” language version questionnaires which were adapted from EDHS and FAO and reviewed from the existed literature, two lab technicians to determine the Hb level, four clinical nurses data collectors, and two BSC nurse supervisors. The questionnaire was prepared in English and then translated into the languages of both Afaan Oromo and Amharic version then retranslated back to English by experts to ensure consistency of the instrument. Two days of training were provided by the principal investigator which is focusing on the objective of the study to create a common understanding of the questionnaire administrations, anthropometric measurements, interviewing approach, and ethical issues. A pretest was conducted among 28 adolescents in Agaro town secondary school, which is 42 kilometers (km) from Jimma town. After pretesting, the necessary corrections were made based on the results of the pretest and the reliability test was performed through Cronbach's alpha (0.758) for selected tools.

Standardization was done for anthropometric measurements, the weight scale was calibrated before data collection with a known standardized object weighing 2 kg, and it checked the functionality routine in between and before measuring the respondentʼs weight. The HemoCue Hb301 analyzer was validated before starting the actual data collection in Jimma University Medical Center, which is found in southwest Ethiopia by comparing with the Sysmex Pyramid XT-1800i model which was taken as a golden standard at a facility level to determine different hematological tests including hemoglobin. Hemoglobin measurement took place at a time with the same sample for 20 individualsʼ blood samples in both machines and the relationship of them were compared through the Pearson correlation coefficient (*r* = 0.985) and coefficient of variation (CV = 0.44).

Data coding and cleaning were performed by cross-checking the printout data for obvious errors. Missing values and outliers were checked before analysis by running descriptive statistics. Supervision was carried out throughout the data collection period both by the supervisors and by the principal investigator to keep the quality of data correctly completed.

#### 2.3.1. Dietary Diversity Score (DDS)

It was assessed in adolescent girls consuming 9 food groups over 24 hours which are starch (cereals and white roots), vegetables, fruits, fish, tubers, meat (including organ meat), milk, egg, and legumes. Each food group had been counted only once resulting in a possible score of 0 to 9. In this study, food groups are categorized into low dietary diversity (≤3 food groups), medium dietary diversity score who consumed four and five food groups, and high dietary diversity (≥6) [17, 18].

#### 2.3.2. Anthropometry

Weight was measured by an electronic digital weight scale (Secca Germany) with minimum/lightly/clothing and no shoes/jewelry and recorded to the nearest 0.1 kg. Calibration was done every morning, and before every weight measurement, the data collectors assured the scales reading exactly at zero. The weight scale was validated against known object weighing 2 kg measured regularly. The same measurer was employed for a given anthropometric measurement to avoid variability after intensive training was given. All measurements had been taken twice, and the average was computed. Similarly, height measurements were carried out using a wooden height measuring board with a sliding head bar. The subjects were asked to stand straight on the leveled surface with heels together and their heads positioned and eyes looking straight ahead (frankfert plane) without shoes. Heals, buttocks, and shoulder blades should touch the vertical surface of the stadiometer. The moving headpiece of the stadiometer was applied to lower to rest flat on the top of the head, and reading was near to 0.1 cm [19].

#### 2.3.3. Knowledge

Anemia-related knowledge was assessed by using a pretested questionnaire. Adolescents in this study have been interviewed on anemia-related knowledge questionnaire that had been adapted from assessing KAP FAO guidelines. It had consisted of eight questions: how can recognize someone who had anemia, consequence of anemia for infants and children, causes of iron deficiency anemia, consequence of anemia in pregnant mother, prevention methods of anemia, sources of iron rich foods, foods increase iron absorption, and foods decrease iron absorption. Then, if the respondents answered the correct answer coded as “1” and “0” if they give the wrong answer regarding anemia. The maximum attainable score was 8 and the minimum possible score is zero. The answers to each question changed to a percentage. An individual who scored 50% and above was considered to have good knowledge and adolescent girls who scored below 50% have been considered to have poor knowledge [20].

#### 2.3.4. Wealth Status

26 items used to assess household fixed assets. The tool was adapted from the Ethiopian demographic and health survey (EDHS), and it was ranked as tertile (low, medium, and high).

#### 2.3.5. Hemoglobin

A portable battery-operated HemoCue Hb 301+ analyzer was used to measure the hemoglobin. It is used mainly in primary care and blood donation setting; it is a simple and convenient solution in poor resource settings. A sample of capillary blood was collected from the ring finger using lancet under strict aseptic precaution, using the thumb, lightly pressing the finger from the top of the knuckle towards the tip. This stimulates the blood flow towards the sampling point. The first drop of blood was wiped away, and the second drop was used for Hb determination. One microcuvette is used only once per individual. After that, the microcuvette was put in the haemoglobinometer, and the reading was observed within 1 minute. Then, reading was classified as normal hg ≥ 12 g\dl and anemic if the hemoglobin value <12 g/dl based on the WHO 2011 recommended cutoff points after adjustments to altitude was made. Adjusted Hb (hemoglobin) concentration was made calculated as Hb = −0.32 *∗* (altitude in meters *∗* 0.0033) + 0.22 *∗* (altitude in meters *∗* 0.0033) 2 for an average altitude of the Jimma town (1780 m) above sea level to subtract (which was 0.56 gm/dl) the adjustment from the individual measured Hb concentration values [7].

### 2.4. Data Processing and Analysis

The collected data were checked for completeness and consistency by manually before entry into a computer. Then, the questionnaires were coded, and data was entered into Epidata version 3.1. Then, the data were exported to SPSS for windows version 20 for further analysis.

Height and weight are transferred into WHO Anthro plus considering the age to convert nutritional data into *Z*-score of indices HAZ and BAZ using the standard of WHO 2007 growth reference. According to this reference, if adolescent girls had BAZ < −2SD, it is considered as thinness, normal if BAZ between −2SD and +1, and overweight was considered as if the 1SD < BAZ ≥ +2SD, whereas if the respondents HAZ ≤ −2SD, it indicates stunting [21].

Descriptive statistics like table and pie charts were used to present data. Frequencies and Percentages were used to organize the categorical independent variables, mean/standard deviation was used to describe a continuous variable, and cross tabs were done to identify adolescents with and without anemia and the prevalence of anemia by its severity.

Both bivariate and multivariable logistic regression analyses were employed to identify the candidate variables and contributing factors of anemia in adolescent girls, respectively. Binary logistic regression analysis was used to identify the candidate variables for multivariable logistic regression at *p* value ≤ 0.25. Adjusted odds ratio (AOR) with 95% CI was used to determine the predictors of the outcome variable independently and to show the strength of an association; *p* value less than 0.05 was considered as statistically significant.

The household wealth status was computed using the PCA method by considering locally available household assets, which were dummy coded. Before running the PCA, assumptions were checked: sampling adequacy with KaiserMeyerOlkin and the results of each analysis was >0.5; presence of substantial correlation was checked by correlation matrix, which showed more than two items has a correlation coefficient >0.3. In addition, Bartlettʼs test of sphericity was checked, and it was significant at *p* < 0.05. After the entire checkup from the total 26 variables, 18 variables with six components were left. Finally, the household wealth status was computed and ranked into three categories after checking chi-squared assumptions.

### 2.5. Ethical Consideration

Ethical clearance was obtained from the Ethical Review Board of Jimma University, Institute of Health, College of public health and medical science, and we also got permission from Jimma Zone Education Department, Jimma zone health office, Jimma town health office, Jimma town administrative education office, school directors, and Keble units. Capillary blood collection was performed after obtaining a signed written informed consent from parents for adolescent girls less than 18 years of age and oral assent from the girls. Girls who are 18 years and above signed a written informed consent form. The aim of the study was explained to all students. Each study participants was informed about the right to withdraw the consent and stop participation at any time without any form of prejudice. Privacy and confidentiality were maintained at each step of the study process. Aseptic techniques were assured by wearing gloves during blood collection and using new lancet for finger pricking. Penetrating injuries were avoided by using fresh self-retractable lancets for all participants to draw a minimal drop of blood for the anemia testing.

The sample blood was never used for further investigation other than hemoglobin analysis and subjects were informed accordingly. Adolescent girls who have anemia were given nutritional education and were asked to visit the nearest health facility.

## 3. Results

### 3.1. Socioeconomic and Demographic Characteristics of Adolescent Girls in Jimma Town, Oromia, Southwest Ethiopia

Five-hundred twenty-eight adolescent girls were interviewed in this study and yielded a 95.6% response rate. The mean ages with a standard deviation of the respondents were 16.48 ± 1.17 years. The majority (89.2%) of respondents was from government schools, and 224 (40.3%) students were from grade 9. More than half (52.8%) of the respondents belong to the Oromo ethnic group. Two-hundred nineteen (41.5%) of the respondents were orthodox by religion followed by Muslims (179 (33.9%)). The majority of (498 (94.3%)) of the adolescent girlsʼ marital status was single. Three-fourth (394 (74.6%)) of the adolescent girls are living with their parents. The majority (80.1%) of the respondentʼs parents were alive. Regarding the parental level of education, more than one-fourth (151 (28.6%)) adolescent girlsʼ fathers had finished grade 12 and above followed by secondary school (grade 9–12) (22.5%). Similarly, one-fourth (25%) of girlʼs mothers were above grade 12 by education and 12.5% of adolescent girlʼs mothers were not able to read and write. Similarly, as of the parental occupational status, 232 (43.9%) of mothers were housewives, whereas 26.9% of mothers were government employees. Forty-three percent of fathers of adolescent girls were government employees, and 29.2% of them were merchants. More than one-third (35%) of adolescent parents were under low economic class (Table 1).

### 3.2. Prevalence of Anemia among Secondary School Adolescent Girls in Jimma Town

**Figure 1:**
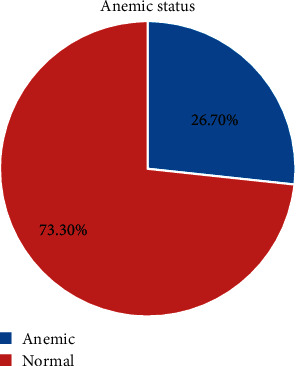
The prevalence of anemia among secondary school adolescent girls in Jimma Town, southwest Ethiopia.

The prevalence of anemia among adolescent girls was 141 (26.7% (95% CI: 22.70, 30.5)) (Figure 1), of which 16.3% were mildly and 10.4% were moderately anemic. No girls reported having severe anemia. The hemoglobin level of the adolescent girls ranged from 8.34 g/dl to 16.84 g/dl with a mean (±SD) value of 13.04 ± 1.70 g/dl.

Seventy-three (52%) of adolescent girls who had anemia were in the age group of 17–19 years, whereas 68 (48%) of them were in the age group 14–16 years old. Similarly, from the total anemic adolescent girls, 48.9% of them live with their family followed by 29.8% of girls who live away from their family and the rest 21.3% of them living with relatives. The proportion of anemia among adolescent girls was 61% and 23.9% among girls who were thin and stunt, respectively.

### 3.3. Knowledge of Adolescent Girls Related to Anemia

**Figure 2:**
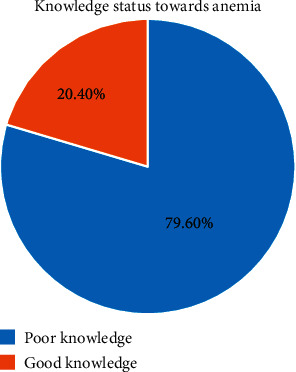
Knowledge status towards anemia among secondary school adolescent girls in Jimma Town.

Three-hundred seventy-one (70.3%) girls heard about iron deficiency anemia. From the total anemic adolescent girls, about 53.2% of girls heard or have information about anemia. The majority of 302 (82.7%) of them responded to have less energy/weakness to recognize somebody has anemia. One-hundred sixty (43.0%) of adolescent girls responded that lack of iron in the diet or eating too little was the cause of iron deficiency anemia followed by heavy bleeding during menstruation 117 (22.2%). Out of 371 respondents who heard about anemia, 296 (79.6%) study participants had poor knowledge related to anemia (Figure 2).

### 3.4. Dietary Diversity Practice of the Adolescent Girls

**Figure 3:**
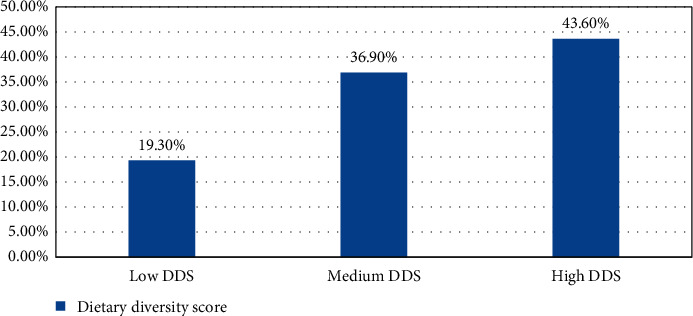
Distribution of DDS among secondary school adolescent girls in Jimma town, southwest Ethiopia.

Regarding DDS, the mean dietary diversity score of the adolescent girls was 5.15 (±1.99). Two-hundred thirty (43.6%) girls had a high dietary diversity score of six and above food groups followed by 195 (36.9%) adolescent girls who had medium dietary diversity scores who consumed four and five different food groups, and the rest respondents had low DDS of three and below food groups. The minimum dietary diversity score of adolescent girls was 1 (consumed only one food group), and the maximum dietary diversity score was 9 out of nine food group items (Figure 3).

### 3.5. Health and Anthropometric Characteristics of Adolescent Girls

Almost all 519 (98.2%) adolescent girls had attained menarche with a reported age of menarche ranging from 9 years to 16 years with a mean age of 14 years of menarche. Out of five-hundred nineteen girls, 57 (10.7%) were on menstruation during data collection time. Four-hundred twenty-three (81.5%) girls changed their pads three and below three times per day. A small number of adolescent girls had a history of malaria infection within one month. Ninety-two (17.4%) girls had a history of worm infestation within one month during data collection time, and 83 (15.7%) of adolescent girls had taken deworm medicine within one month (Table 2).

The mean height and weight of the adolescent girls were 157.3 (±6.056) cm and 50.66 (±7.35) kg, respectively. The mean body mass index for age *Z* score was −0.259 (±1.0775), and the mean height for age *Z* score was −0.7690 (±0.895), respectively. Thinness was recorded in 41 (7.8%) of which 10 (1.9) were severed thinness. Sixty-one (11.6%) of girls were overweight, of which only a few individuals 4 (0.8%) were obese. Stunting which is the chronic form of malnutrition is seen in 46 (8.7%) adolescent girls in the study area.

### 3.6. Factors Associated with Anemia

The motherʼs educational status, living condition of the adolescent girls, dietary diversity score, and duration of menses, meal frequency, and wealth index were found to be a significant association with anemia by multivariate logistic regression at *p* value <0.05.

The odds of having anemia were 4.4 times higher among girls who lived separately from their parents compared to girls live with their parents (AOR = 4.43 (95% CI, 2.20, 8.90), *p* < 0.001)). Similarly, the odds of having anemia were 3.6 times higher among adolescent girls who had low DDS compared with girls who had high DDS (AOR = 3.57 (95% CI, 1.88, 6.76, *p* < 0.001)). Additionally, the odds of having anemia were 2.2 times higher among adolescent girls having a duration of menses greater than five days compared with girls who had a duration of menses less than or equal to five days (AOR = 2.25 (95% CI, 1.17, 4.33, *p*=0.028). Adolescent girls who were from low-income families were 2 times more likely to be anemic compared with girls who had high-income families AOR = 2.16 (95% CI, 1.17, 3.99, *p*=0.002). However, maternal educational status of having at least attended a secondary school was negatively associated with anemia among adolescent girls compared with mothers who cannot read and write AOR = 0.43 (95% CI, 0.19, 0.98, *p*=0.016) (Table 3).

## 4. Discussions

The study was attempted to assess the magnitude of anemia and associated factors among adolescent girls in Jimma town high schools. The result of this study indicated that the overall prevalence of anemia among adolescent girls was 26.7% (95% CI, 22.7–30.5). According to WHO criterion, if the prevalence of anemia was within 20% to 39.9%, it is considered as a moderate public health concern, so anemia in adolescent girls in Jimma town is a moderate public health concern. Among adolescent girls who had anemia, the magnitude of mild and moderate anemia was 61%, and 39%, and no one was reported to have severe anemia. The factors associated with having anemia among adolescent girls include mother education, duration of menses, low dietary diversity score, living condition of adolescent girls, and lower economic class of the family.

This finding is in line with that reported in Kenya (26.5%) [15], the local study reported from Filtu town Somali region (23.66%) [11], consistent somehow in Berhale district afar region (22.8%) [13], Dembia, northwest Ethiopia (25.5%) [22], from research done in three districts of Ethiopia, namely, Debrelibanose, Laygayint, and Damotegale, an average prevalence of anemia was 29% [23].

However, the finding of this result is found to be lower than that of studies conducted in Nigeria which showed the overall prevalence of anemia of 47.5% [24]. The possible reason could be that a low proportion of (1.7%) of adolescents consumed organ meat like that of the current study (12.3%). Similarly higher prevalence of anemia was reported in Lahore, Pakistan, where about 68.9% of participants were anemic, this might be because about three-fourth (77%) of the participants did not have green vegetables in their diet like that of the current study which was 28.2% [25]. The finding of the current study was lower than that of the study reported in Nepal, where the overall prevalence of anemia was 42.5%; the possible reason might be 42.4% of adolescent girls had a history of worm infestation as compared with the current study 17.4% [26].

The finding of this study was higher than that reported in central Kerala, India, where the overall prevalence of anemia was 21%; the possible reason might be more than half (56.25%) of the respondents were taken deworm medication like that of the current study (15.7%) [27]. Similarly, the current study result was higher than the finding reported in Turkey where the overall prevalence of anemia among adolescent school girls was 8.3% [28]. This could be due to differences in socioeconomic and cultural behaviors including dietary habit differences.

The result of this study is also higher than that conducted in the local studies, Bahirdar, northern Ethiopia, where the overall prevalence of anemia was 11.1% [29]. The difference might be because nearly half of adolescent girls had a medium dietary diversity score as compared with that of our study (36.9%). A similar study performed in the Kebena Guraga zone, southwest Ethiopia, revealed that the overall prevalence of anemia was 12% and Mekelle 11% which is lower than that of the current finding [30, 31].

In addition to the abovementioned factors, to make difference regarding the magnitude of anemia among adolescent girls could be due to differences in sociocultural and behavioral practice including dietary habit differences between one another in the world. The result of our study also varied with the result of local studies; it might be because the differences of the study period could lead to seasonal variation.

Adolescent girls whose mothers attended at least secondary school were 59.6% less likely to develop anemia than adolescent girls whose mothers cannot read and write. Similar studies were reported in four populous villages of India and the urban slum of Kanpur, Uttar Pradesh, India, adolescent girls whose mothers were either illiterate or had only primary education developed anemia than their counterparts [32, 33]. This is in contrast with the finding of the study in Nepal [26] and Kenya [15] where mother's educational status did not show significant association with anemia among adolescent girls. The possible justification is that there is a difference in the study area and sociocultural factors regarding the education and feeding habits of the children and adolescents. In our context, it gives a high sense that when women are more educated, they will have knowledge regarding a balanced diet and know the sources of nutrients especially in this case iron sources of foods and know how to give care to their children. In addition to this, as we know, most of the time, mothers collected a variety of foods which are used for eating purpose at home and knowledge might be required in order to incorporate foods that contain iron-rich foods.

The odds of having anemia were 4.4 times higher among adolescents living separately from their family than those lived with the family. Similarly, the odds of having anemia were 2.5 times higher among adolescents who lived with their relatives compared with those who lived with their families. A similar finding was reported from a study conducted in Dembia, northwest Ethiopia, where school going adolescent girls who lived with their guardians are more affected by anemia compared with their counterparts [22]. The possible reason might be, out of the adolescent girls who lived separately from their family, about 59.6% of adolescent girls had low dietary diversity scores and 63.1% were from low household wealth status. Similarly, adolescent girls who lived with relatives (44.9%) of their families were under low wealth status. This is because the economic status has significant implications in their purchasing power in order to get a balanced diet.

The present study also tells the odds of having anemia were 3.5 times higher among adolescents whose DDS is low compared with those who have high DDS. A similar study was reported from India, Nepal, Nigeria, and Kenya as consumption of low diversified foods was associated with anemia among adolescent girls [15, 24, 27, 34]. It might be the dietary diversity that tells the number of variety of food groups consumed over 24 hours prior to the data collection period is widely recognized as a key dimension of diet quality in individuals and households. Diet diversity is strongly associated with nutrient adequacy including iron adequacy [17].

This finding was also consistent with the report of the study result conducted in Tigray, north Ethiopia [10] and Dembia district, northwest Ethiopia [24], and this might be due to socioeconomic similarity. This means that adolescents who consumed less quality diet are more likely to be anemic since the probability of nutrient adequacy increases as diet variety or diversity increases.

In this study, the history of worm infestation for the last one month before data collection was not significantly associated with anemia among adolescent girls; this is not in agreement with a similar study in Bahirdar where adolescent girls with a history of parasitic infection were 2.8 times more likely to be anemic than those without intestinal parasite [29]. This study is also not consistent with the study result reported in Kenya where the adolescent girls who had a history of worm infestation were 12 times more likely to be anemic than those who did not have a history of worm infestation [15]. This variation might be because the number of girls with a history of worm infestation in our study is small in number that could lead to the regression to generate no association between worm infestation and anemia. The second reason might be because girls who are actually infected with an intestinal worm are missed and reported as not having worm infestation as this study is limited to history taking rather than laboratory stool examination.

The history of malaria infection for the last one month before data collection period also was not significantly associated with anemia in this study which is in contrast with the study result reported in Kenya where the presence of malaria parasitemia was 3.8 times more likely to be anemic than who did not have malaria parasitemia [15]. The reason for this study not having an association with anemia may be smaller number of cases reported to have a history of malaria, and this also might be because the history was collected verbally from the participants instead of any test of blood, and this might have masked the actual status of the respondents. Another possible reason might be our country Ethiopia had implemented a mass campaign distributed nearly 29.6 million long-lasting insecticidal nets (ITNs) to protect all Ethiopians living in areas with ongoing malaria transmission through an expansion of health extension workers [35].

The odds of anemia were 2 times higher among adolescents who had menstrual bleeding more than 5 days as compared with adolescents girls with menstrual bleeding less than or equal to 5 days. This finding was consistent with the study result reported in the Tang ail region of Bangladesh, central Kerala, India, Nepal, and locally Bahirdar, northwest Ethiopia [27, 29, 34, 36]. This might be due to the fact that with an increased duration of menstruation, there will be a high chance of more blood loss that can lead to anemia.

This study revealed that low economic status has been one factor for anemia in adolescent girls (AOR = 2.684 (1.457, 4.945)). The odds of anemia were 2.6 times higher among adolescents whose families are under low wealth status than those who had high wealth status families. This finding is also comparable with the study report result conducted in the Berhale afar region [37]; in this study, adolescent girls with low economic status were 2.8 times more likely to be anemic compared with counterpart. This finding is also consistent with that conducted in the Tang ail region of Bangladesh [36], Amravati city, India, and Chennai, Tamil Nadu, India [38]. This implies that, in low family income, it is difficult to obtain a variety of foods including iron-rich food sources.

## 5. Conclusion and Recommendation

In this study, the prevalence of anemia was a moderate public health problem. Factors associated with anemia were low wealth status, adolescent girls living separately from their parents, low dietary diversity score, and duration of menses greater than five days. However, adolescentsʼ mother who had attended at least secondary school was a protective factor for anemia in adolescent girls in this study.

## Figures and Tables

**Table 1:** Socioeconomic and demographic characteristics of adolescent girls and their parents in Jimma town, Oromia, southwest Ethiopia.

Variables (*N* = 528)	Categories	Frequency (*n* = 528)	Percent (%)
Age	14–16	274	51.9
	17–19	254	48.1

Ethnicity	Oromo	279	52.8
	Amhara	100	18.9
	Guraga	42	8.0
	Dawuro	28	5.3
	Keffa	23	4.4
	Yeme	30	5.7
	Others^1^	26	4.9

Religion	Orthodox	219	41.48
	Muslim	179	33.9
	Protestant	117	22.16
	Catholic	13	2.46

Family size	≤5	239	45.3
	>5	289	54.7

Marital status	Single	498	94.3
	Married	30	5.7

Parental condition	Parents alive	423	80.1
	Only father alive	47	8.9
	Only mother alive	40	7.6
	Dead	18	3.4

Living status of adolescent girls	Living with parents	394	74.6
	Living with relatives	69	13.1
	Living separately from family	65	12.3

Maternal educational status	Cannot read and write	66	12.5
	Can read and write	95	18.0
	Primary (1–8)	117	22.2
	Secondary (9–12)	118	22.3
	Above 12	132	25.0

Father educational status	Cannot read and write	29	5.5
	Can read and write	119	22.5
	Primary (1–8)	109	20.6
	Secondary (9–12)	120	22.7
	Above 12	151	28.6

Maternal occupation	House wife	232	43.9
	Merchant	91	17.2
	Government employee	145	26.9
	Private employee	48	9.1
	Daily laborer	12	2.3

Father occupational status	Government employee	228	43.2
	Merchant	154	29.2
	Daily laborer	18	3.4
	Private employee	103	19.5
	Farmer	25	4.7

Wealth status	Low	185	35
	Medium	172	32.6
	High	171	32.4

Others^1^, Tigray, Silte, Wolayita.

**Table 2:** Health-related characteristics of the study participants among adolescent high school girls in Jimma town, Oromia, southwest Ethiopia.

Variables	Category	Frequency	Percent
Status of menarche	Attained	519	98.3
	Not attained	9	1.7

Age at the onset of menarche (*N* = 519)	≤14	412	79.4
	>14	107	20.6

Duration of menses in each cycle (*N* = 519)	≤5 days	452	87.1
	>5 days	67	12.9

Frequency of changed pad/day (*N* = 519)	≤3 times	427	82.3
	>3 times	92	17.7

Had menstruation currently (*N* = 519)	Yes	57	10.7
	No	462	89.3

History of malaria (*N* = 528)	Yes	51	9.7
	No	477	90.3

History of worm infestation (*N* = 528)	Yes	92	17.4
	No	436	82.6

Taking deworm medicine (*N* = 528)	Yes	83	15.7
	No	445	84.3

**Table 3:** Binary and multivariable logistic regression model to identify factors associated with anemia among adolescent high school girls in Jimma town, Oromia, southwest Ethiopia.

Variables	Category	Outcome variables		COR 95% CI	AOR 95% CI
		Anemia (%)	Normal (%)		
Father education	Cannot read and write	12 (41.4)	17 (58.6)	1	—
	Can read and write	38 (36.2)	81 (63.8)	0.66 (0.29, 1.53)	0.540 (0.19, 1.56)
	Primary (1–8)	33 (30.3)	76 (69.7)	0.62 (0.26, 1.43)	0.672 (0.28, 1.99)
	Secondary (9–12)	27 (22.5)	93 (77.5)	0.41 (0.17, 0.97)^*∗*^	0.48 (0.16, 1.43)
	Above 12	31 (20.5)	120 (79.5)	0.37 (0.16, 0.85)^*∗*^	0.90 (0.30, 2.70)

Mother education	Cannot read and write	29 (43.9)	37 (56.1)	1	1
	Can read and write	42 (44.2)	53 (55.8)	1.10 (0.54, 1.90)	1.56 (0.73, 3.35)
	Primary (1–8)	36 (30.8)	81 (69.2)	0.57 (0.30, 1.06)	0.85 (0.40, 1.81)
	Secondary (9–12)	18 (15.3)	100 (84.7)	0.23 (0.11, 0.46)^*∗*^	0.43 (0.19, 0.98)^*∗∗*^
	Above 12	16 (12.1)	116 (87.9)	0.18 (0.09, 0.36)^*∗*^	0.44 (0.18, 0.95)^*∗∗*^

Living status	Living with parent	69 (17.50)	325 (82.5)	1	1
	Living with relatives	30 (43.5)	39 (56.5)	3.62 (2.11, 6.23)^*∗*^	2.51 (1.35, 4.67)^*∗∗*^
	Living separately from family	42 (64.6)	23 (35.4)	8.60 (4.86, 15.22)^*∗*^	4.43 (2.20, 8.90)^*∗∗*^

Heard about anemia	Yes	75 (20.2)	296 (79.8)	1	1
	No	66 (42)	91 (58.0)	2.86 (1.91, 4.29)^*∗*^	1.140 (0.66, 1.96)

Meal frequency per day	Once	7 (11.1)	5 (88.9)	4.97 (1.42, 17.35)^*∗*^	1.23 (0.231, 6.62)
	Two times	38 (56.6)	43 (43.4)	3.14 (1.62, 6.07)^*∗*^	1.08 (0.47, 2.49)
	Three times	76 (21.2)	268 (78.8)	1.07 (0.58, 1.76)^*∗*^	0.58 (0.30, 1.09)
	Four and above	20 (22.0)	71 (78.0)	1	

Menstruation status (*N* = 519)	Yes	22 (61.4)	35 (38.6)	1.92 (1.08, 3.40)^*∗*^	1.93 (0.94, 3.94)
	No	114 (73.8)	348 (24.7)	1	1

Thinness	Thinness	25 (61.0)	16 (39.0)	4.99 (2.58, 9.68)^*∗*^	2.31 (0.91, 5.45)
	Normal	116 (23.8)	371 (76.2)	1	1

DDS	Low	59 (57.3)	44 (42.7)	7.73 (4.53, 13.18)^*∗*^	3.57 (1.88, 6.76)^*∗∗*^
	Medium	48 (24.6)	147 (75.4)	1.88 (1.15, 3.07)^*∗*^	1.90 (1.11, 3.27)^*∗∗*^
	High	34 (14.8)	196 (85.2)	1	1

Family size	≤5	43 (18.6)	188 (81.4)	1	1
	>5	98 (33.0)	199 (67.0)	2.15 (1.43, 3.24)^*∗*^	1.24 (0.74, 2.07)

Wealth index	Low	77 (42.5)	104 (57.5)	4.77 (2.84, 8.02)^*∗*^	2.16 (1.17, 3.99)^*∗∗*^
	Medium	39 (22.3)	136 (77.7)	1.69 (0.97, 2.93)	1.24 (0.66, 2.32)
	High	25 (14.5)	147 (85.5)	1	

Frequency of changed pad (*N* = 519)	≤3 times p/day	102 (23.9	325 (76.1)	1	1
	3> times p/day	34 (37)	58 (63.0)	1.87 (1.16,3.01)^*∗*^	1.68 (0.92, 2.97)

Duration of menses (*N* = 519)	≤5 days	100 (21.1)	352 (77.9)	1	
	>5 days	36 (53.7)	31 (46.3)	4 (2.4, 6.9)^*∗*^	2.25 (1.17, 4.33)^*∗∗*^

^*∗*^variables at *p*-value ≤0.25 in bivariate logistic regression, ^*∗∗*^predictor variables in multivariate logistic regression at *p* < 0.05.

## Data Availability

The data that support the findings of this study can be made available from the corresponding author upon reasonable request (e-mail: kelemu.fentie2015@gmail.com).

## Abbreviations

AOR: Adjusted odds ratio
BAZ: Body Mass Index for age, *Z* score
BMI: Body Mass Index
BSc: Bachelor of Science
CI: Confidence interval
COR: Crude odds ratio
CSA: Central Statistics Authority
DALYs: Disability adjusted life years
DDs: Dietary Diversity Score
FANTA: Food and Nutrition Technical Assistance
FAO: Food and Agriculture Organization
Hb: Hemoglobin
IDA: Iron Deficiency Anemia
LMICs: Low- and Middle-Income Countries
MOH: Ministry of Health
NCHS: National Center for Health Statistics
OR: Odds ratio
PCA: Principal component analysis
SD: Standard deviation
SPSS: Statistical package for social science
UNICEF: United Nations International Children Emergency Fund
WHO: World Health Organization.

## Supplementary Materials

Supplementary Materials Annex 1 (schematic sampling procedure).
