# Supplementary material for: Prevalence of Anemia and Associated Factors among Secondary School Adolescent Girls in Jimma Town, Oromia Regional State, Southwest Ethiopia
Source: Anemia. 2020 Sep 22;2020:5043646. doi: 10.1155/2020/5043646 (PMC7528150; doi:10.1155/2020/5043646)
Supplement: Supplementary Materials — Annex 1 (schematic sampling procedure). [file 5043646.f1.docx]

**Annex-1: Schematic representation of sampling procedure, to assess the prevalence of anemia and associated factors among school adolescent girls, Jimma, Ethiopia.**

Simple random sampling was used to select the study participants

**Jimma town high school adolescent girls (N=14)**

From the total 14 schools 30% of them which are five schools were selected by using simple random sampling method

130 adolescent girls G-9= 75 G-10=55

Jiren secondary school

N=1354

Tesfa Tewahido secondary school N=165

Jimma preparatory school

N=770

Catholic secondary school

N= 175

Proportionate allocation was made for each school and grade sections.

Seto semero secondary school

N=758

231 adolescent girls G-9 =131 G-10=100

28 adolescent girls

G-9=17 G-10=11

132 adolescent girls G-11=72 G-12=60

30 adolescent girls G-9=18 G-10=12

Total sample size n=551
